# Supplementary material for: Light- and Redox-Responsive Block Copolymers of mPEG-SS-ONBMA as a Smart Drug Delivery Carrier for Cancer Therapy
Source: Pharmaceutics. 2022 Nov 24;14(12):2594. doi: 10.3390/pharmaceutics14122594 (PMC9788424; doi:10.3390/pharmaceutics14122594)
Supplement: Supplementary file 1 [file pharmaceutics-14-02594-s001.zip › pharmaceutics-2001400-supplementary.pdf]

Supporting Information

Light- and Redox-Responsive Block Copolymers of mPEG-SS-ONBMA as a Smart Drug Delivery Carrier for Cancer Therapy

Yu-Lun Lo,[a#] Yao-Hsing Fang,[a#] Yen-Ju Chiu,[a] Chia-Yu Chang,[a] Chih-Hsien Lee,[a] Zi-Xian Liao[b] and Li-Fang Wang[a,b,c*]

[a]Department of Medicinal and Applied Chemistry, College of Life Sciences, Kaohsiung Medical University, Kaohsiung 807, Taiwan

[b]Institute of Medical Science and Technology, National Sun Yat-Sen University, Kaohsiung 804, Taiwan

[c]Department of Medical Research, Kaohsiung Medical University Hospital, Kaohsiung 807, Taiwan

#These authors contributed equally to this work.

Correspondence to: Li-Fang Wang, Professor of Medicinal & Applied Chemistry

College of Life Sciences
Kaohsiung Medical University
100, Shih-Chuan 1st Rd, Kaohsiung City 807, Taiwan
Tel: 011-886-7-3121101-2217
Fax: 011-886-7-3125339
E-mail: lfwang@kmu.edu.tw

(A)

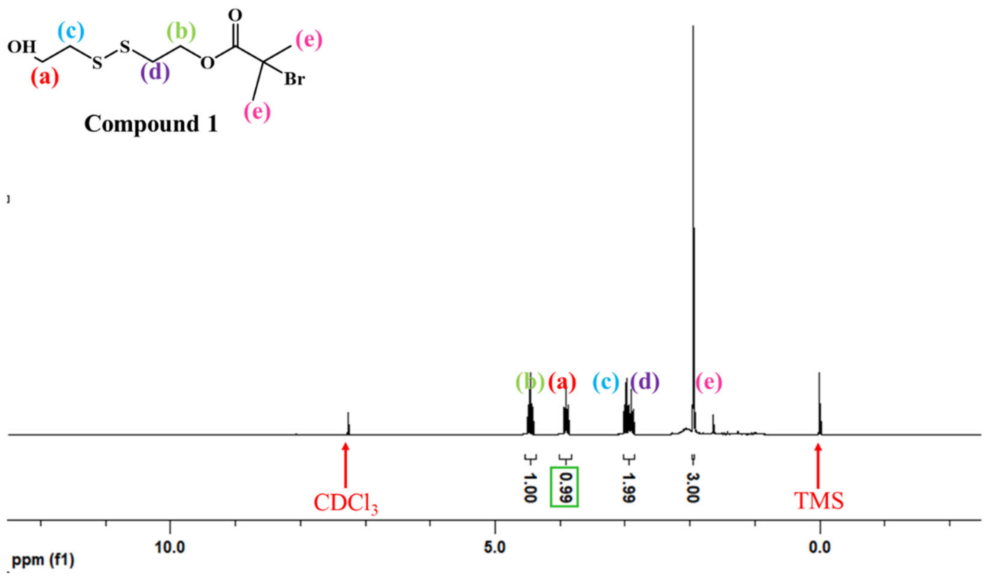

(B)

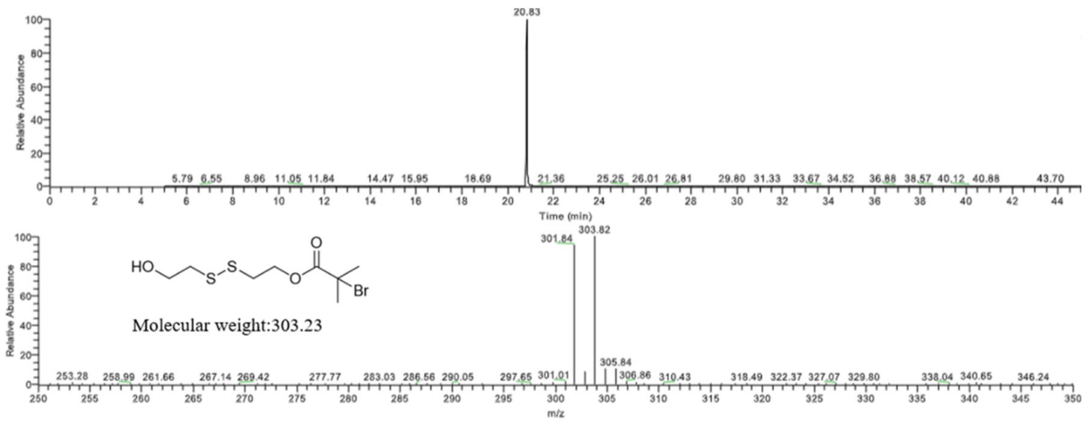

Figure S1. ^{1}H-NMR spectrum (A) and LC/MASS spectrum (B) of 2-hydroxyethyl-2’-(bromoisobutyryl) ethyl disulfide (HO-SS-Br, Compound 1)

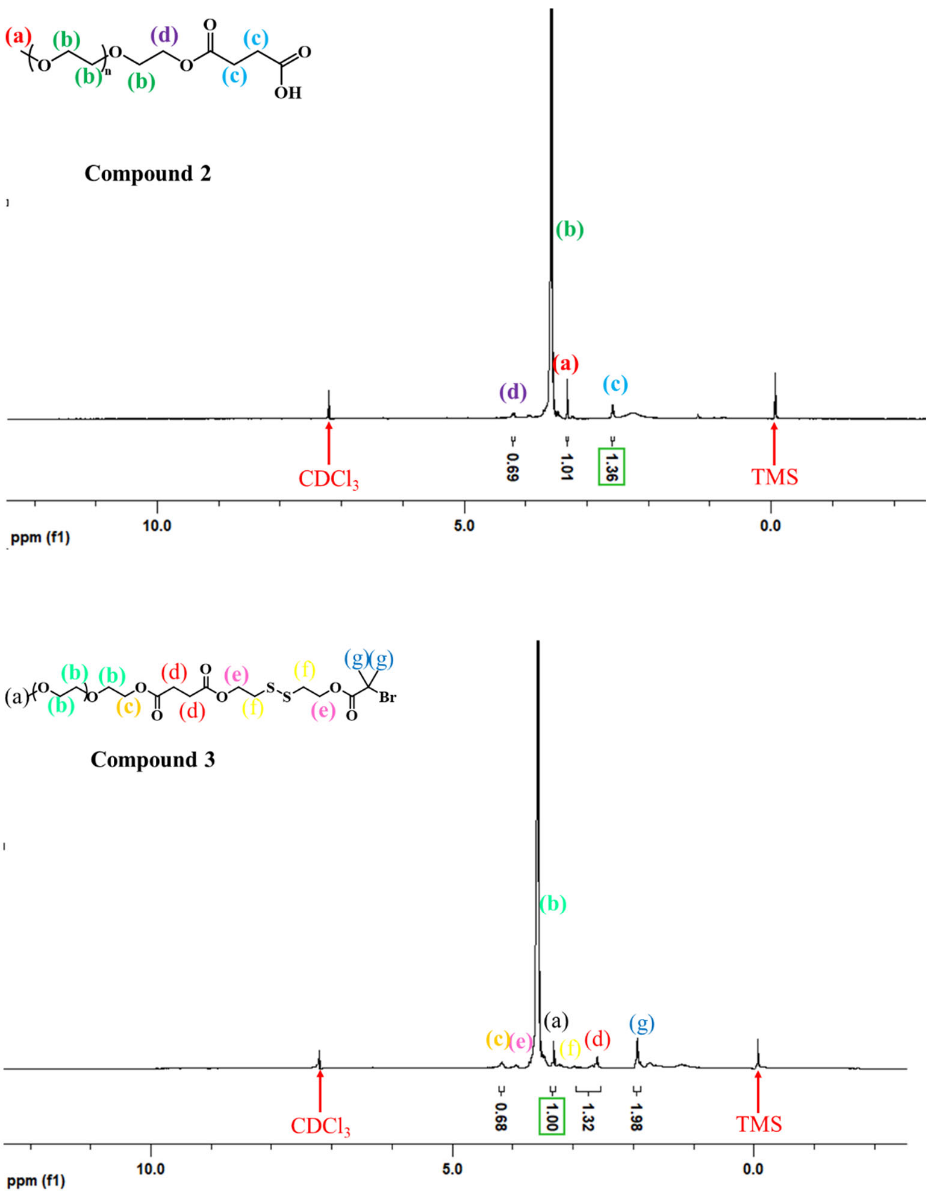

Figure S2. ^{1}H-NMR spectra of mPEG-COOH (Compound 2) and mPEG-SS-Br (Compound 3)

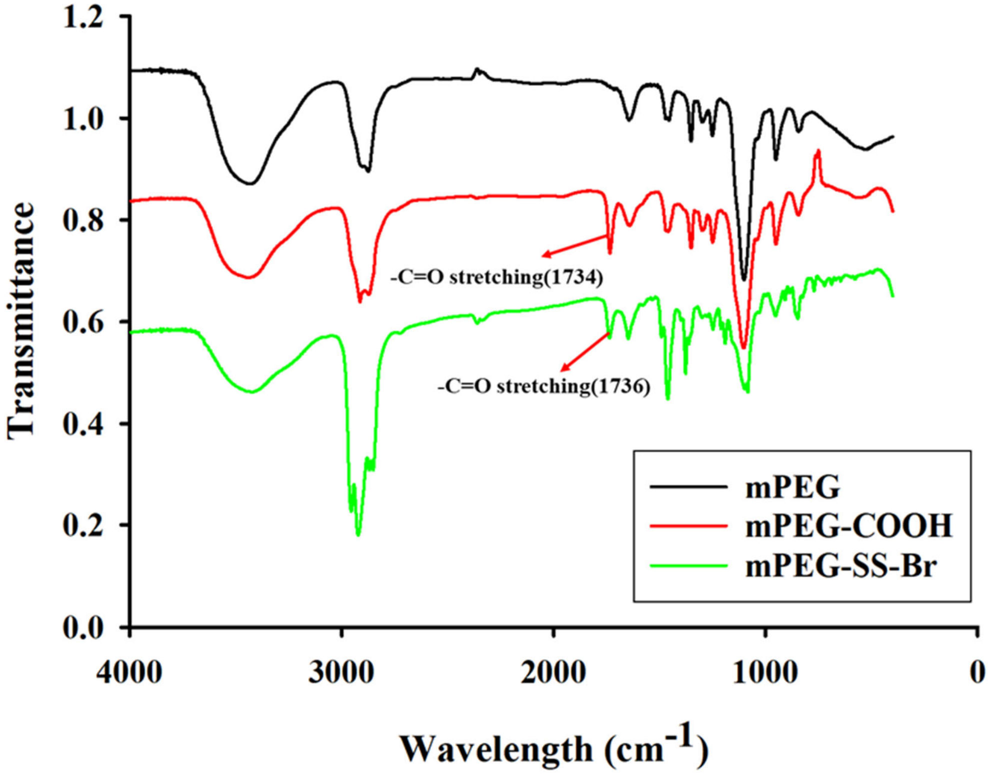

Figure S3. FTIR spectra of mPEG (2000 MW) , mPEG-COOH (Compound 2) and mPEG-SS-Br (Compound 3)

(A)

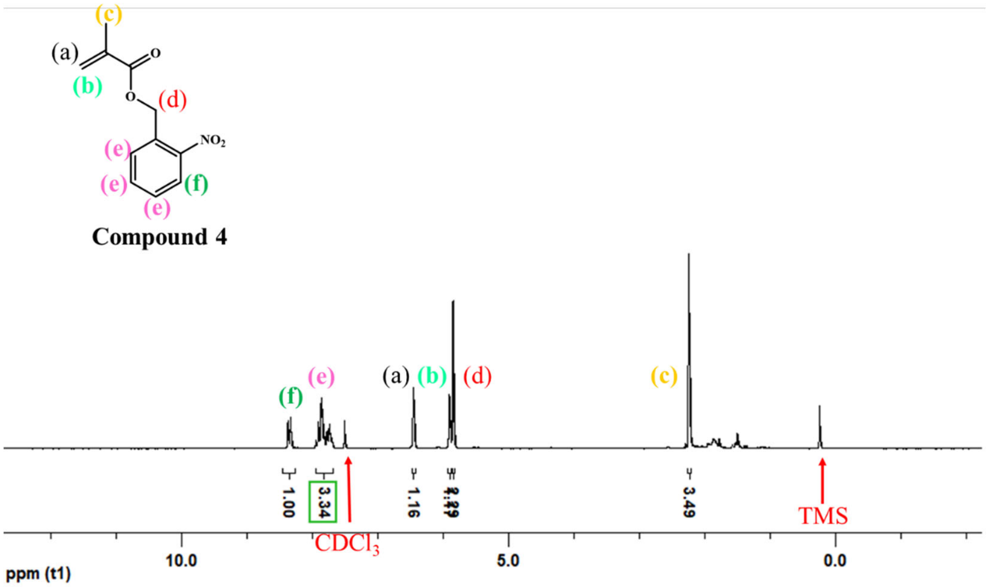

(B)

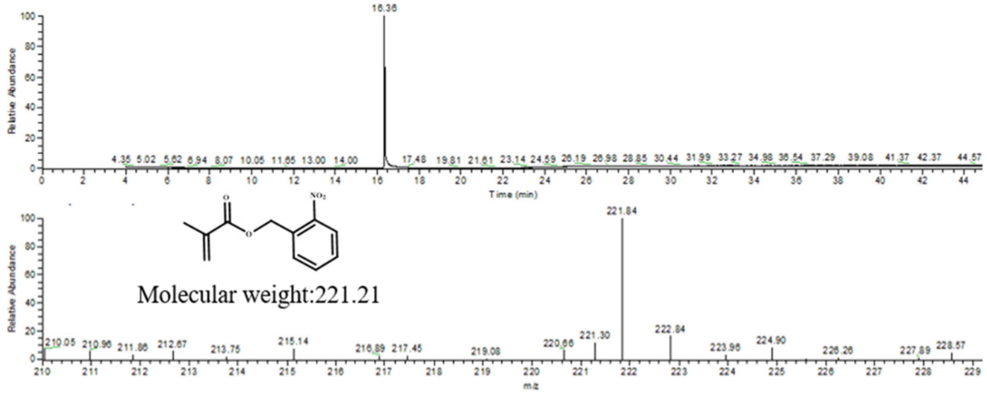

Figure S4. ^{1}H-NMR spectrum (A) and LC/MASS spectrum (B) of o-nitrobenzyl methacrylate (ONBMA, Compound 4)

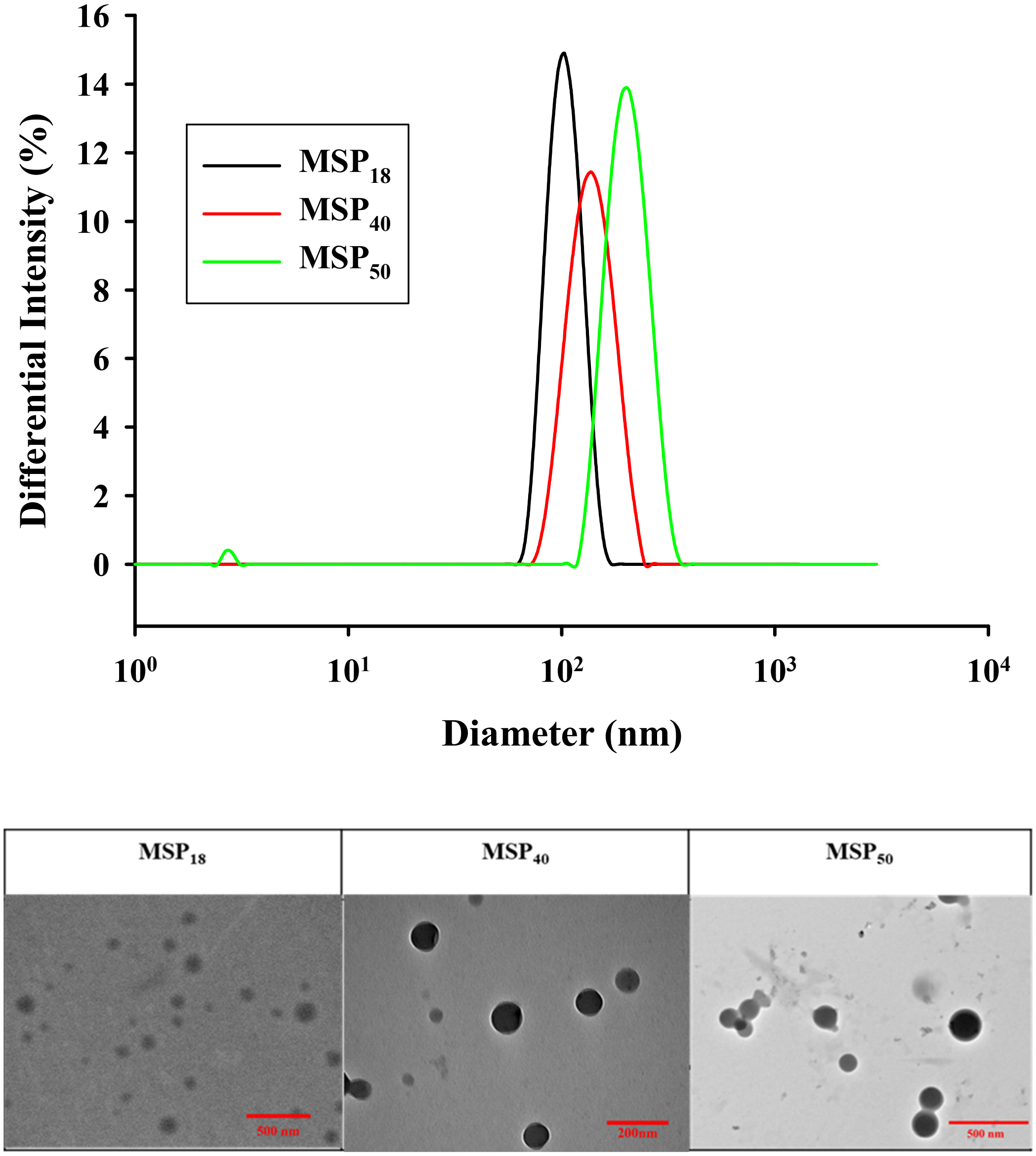

Figure S5. DLS diagrams and TEM images of MSP micelles

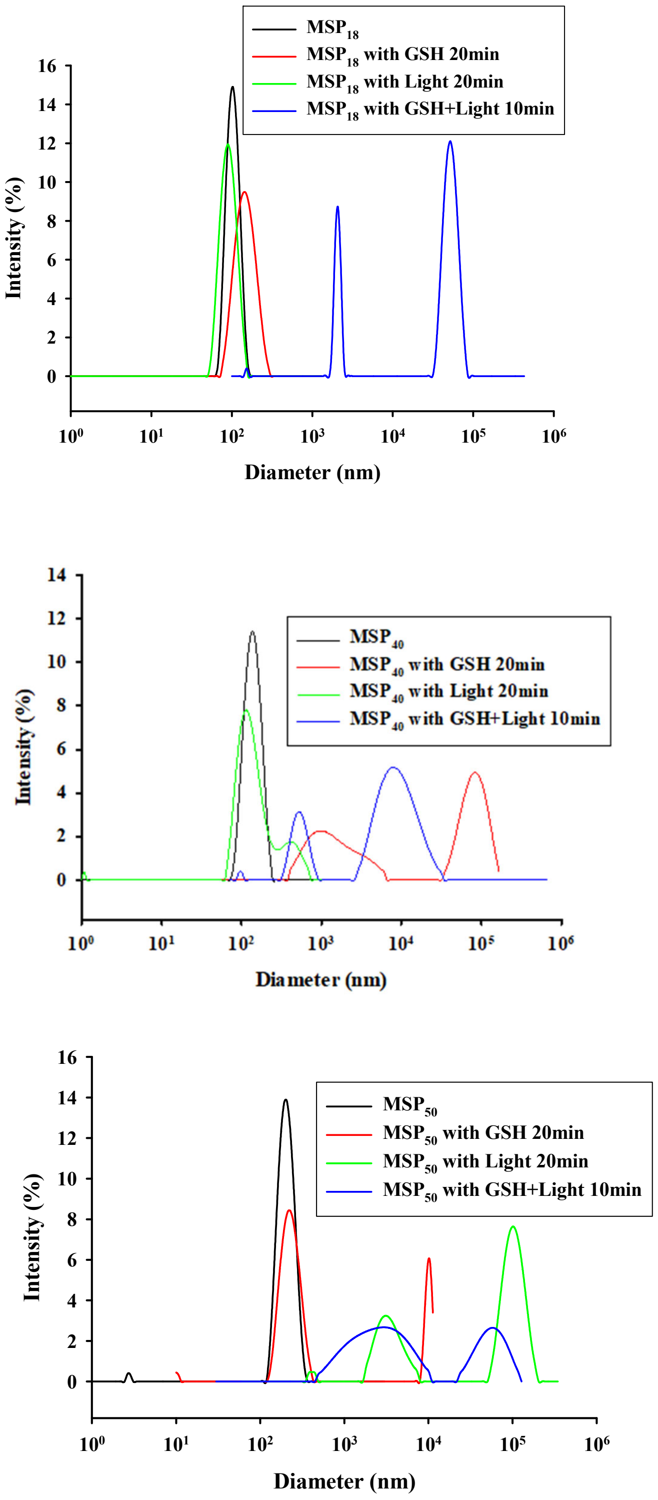

Figure S6. DLS profiles of MSP micelles with different treatments: Control (blank), 5 mM GSH for 20 min (red), UV light for 20 min (green), and the combination of 2.5 mM GSH and UV light for 10 min (blue).

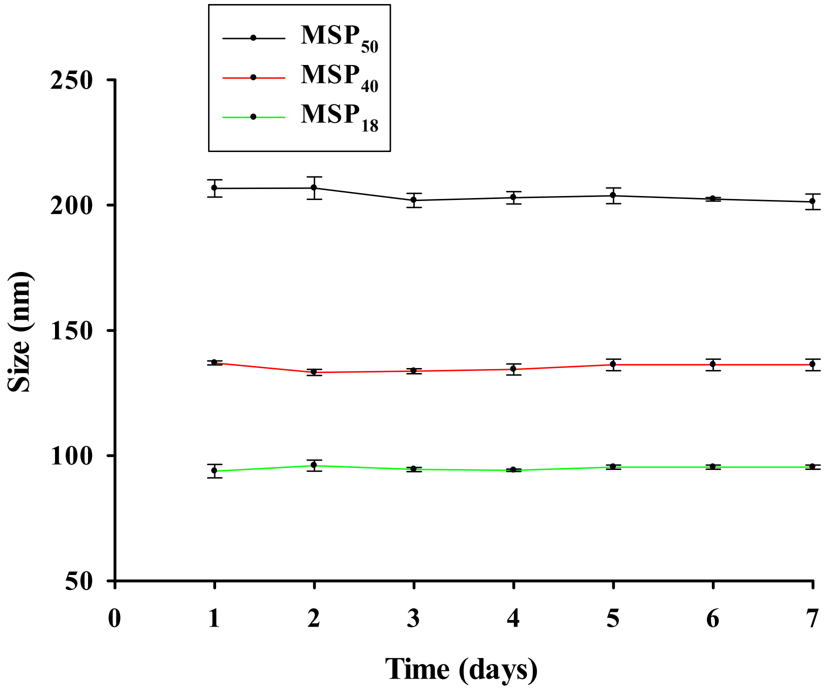

Figure S7. Stability testing of MSP micelles at 0.1 mg/mL in deionized water by DLS.

(A)

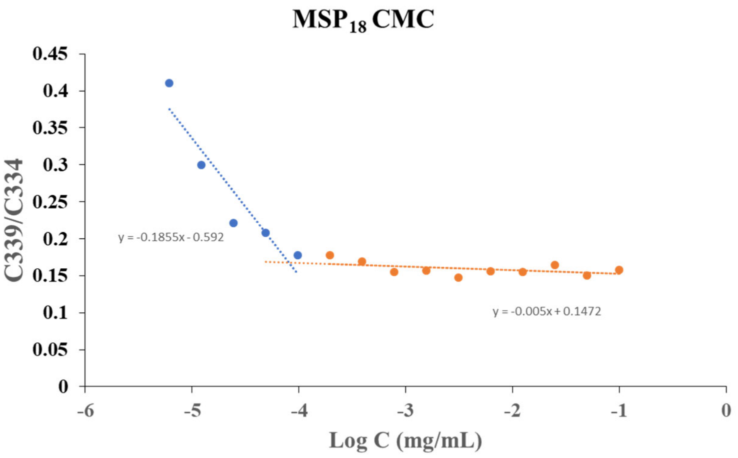
MSP_{18} CMC
C339/C334
y = -0.1855x - 0.592
y = -0.005x + 0.1472
Log C (mg/mL)

(B)

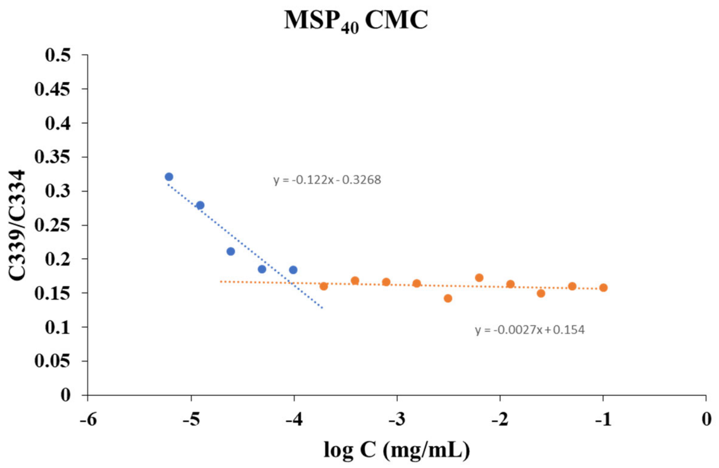
MSP_{40} CMC
C339/C334
y = -0.122x - 0.3268
y = -0.0027x + 0.154
log C (mg/mL)

(C)

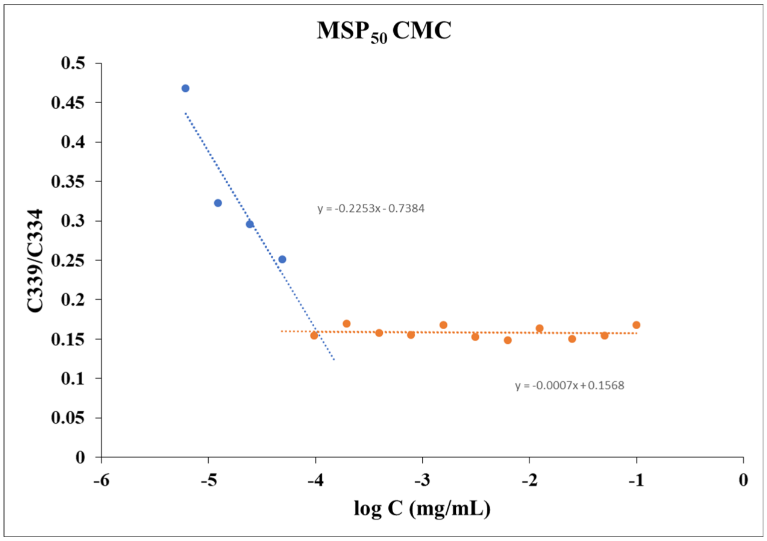
MSP_{50} CMC
C339/C334
y = -0.2253x - 0.7384
y = -0.0007x + 0.1568
log C (mg/mL)

(D)

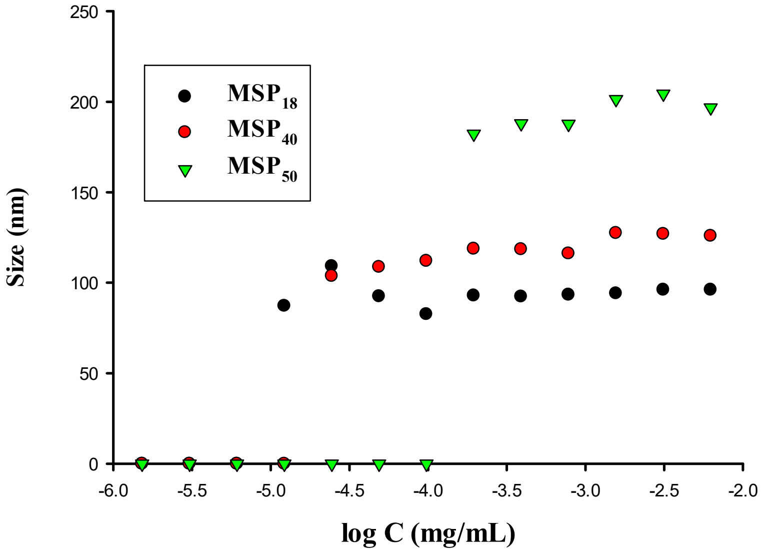

Figure S8. Critical micellar concentrations of MSP_{18} (A), MSP_{40} (B), and MSP_{50} (C) measured by fluorescence spectrometer using pyrene as a probe and by dynamic light scattering (D).

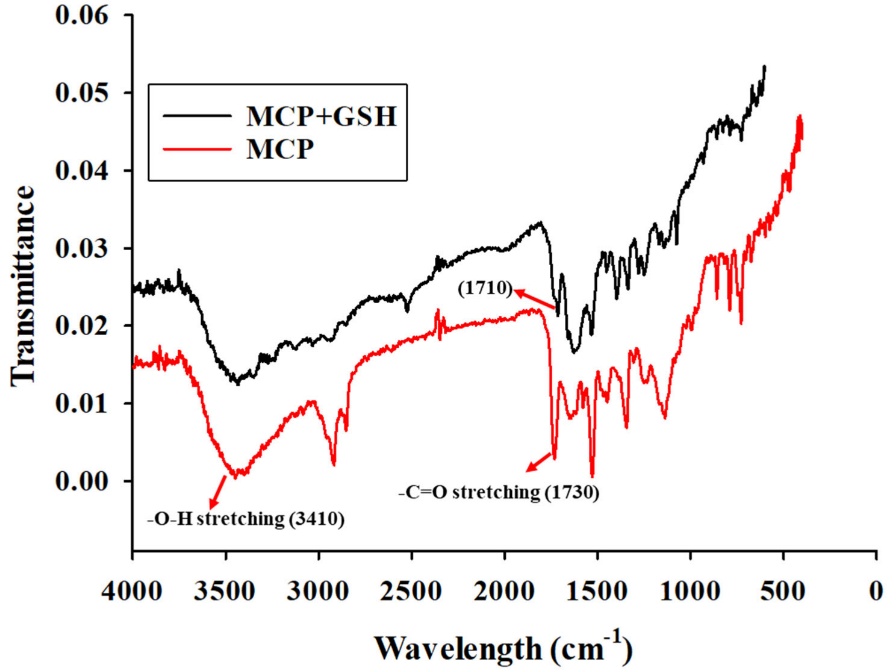

Figure S9. FTIR spectra of the MCP micelle with and without GSH treatment.

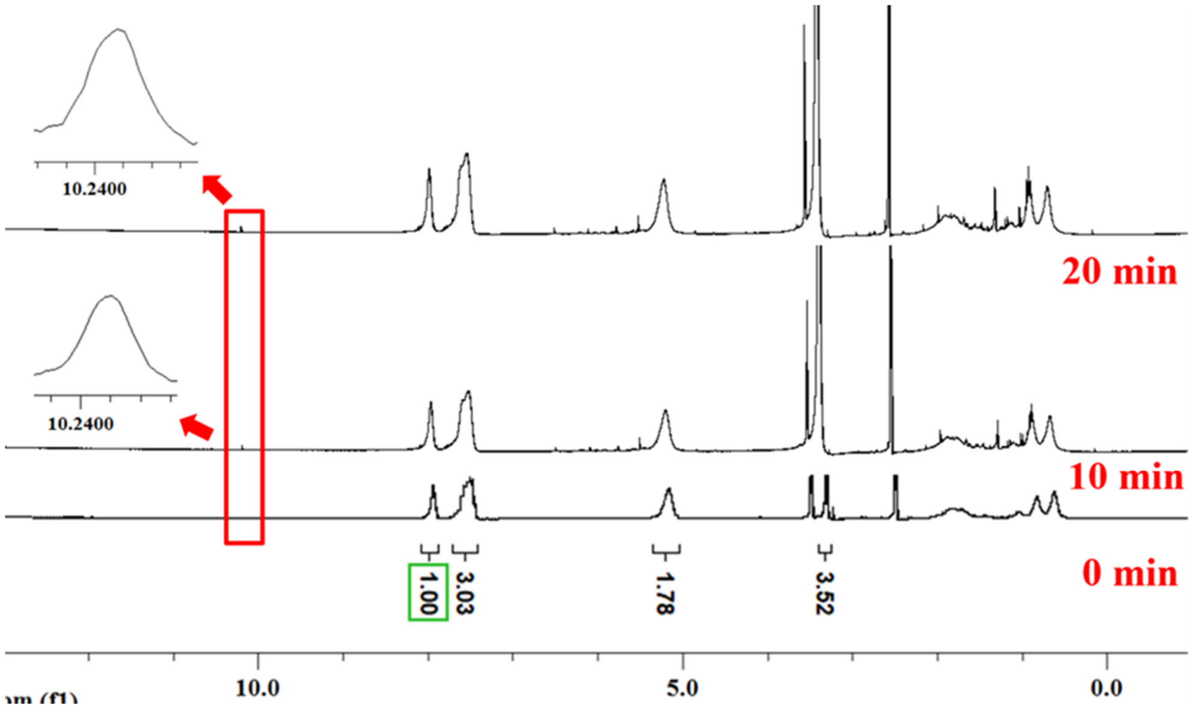

Figure S10. ^{1}H-NMR spectrum of MSP_{50} upon UV light irradiation for 0, 10 and 20 min.

(A)

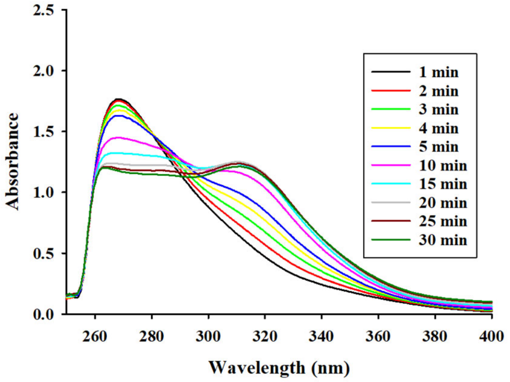

(B)

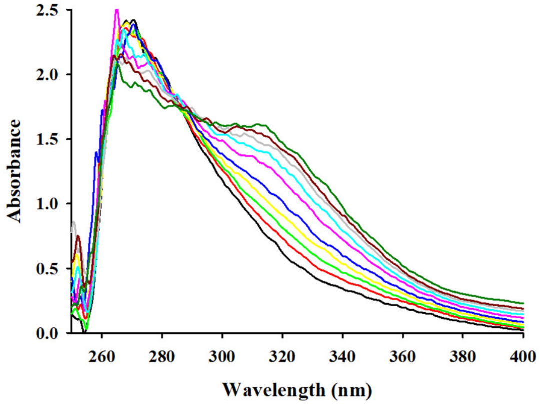

(C)

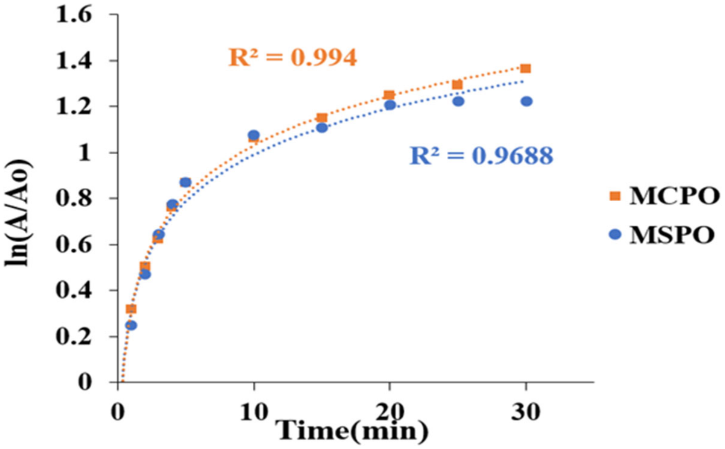

Figure S11. UV-visible spectra of the MSP_{50} (A) and MCP_{50} (B) copolymers at 0.1 mg/mL in DMSO solution upon UV-light irradiation (365nm, 430mW. cm^{-2}) for various durations. (C) The plot of absorbance intensity ratio at 340 nm with time.
